# Effects of diabetes on the development of radiation pneumonitis

**DOI:** 10.1186/s12931-021-01754-4

**Published:** 2021-05-24

**Authors:** Guangtong Dong, Yuxiao Li, Qiyao Zhao, Bing Pang, Xin Qi, Junping Wei, Wei Hou

**Affiliations:** 1 Department of Endocrinology, Guang’anmen Hospital of China Academy of Chinese Medical Sciences, 6 Floors of Inpatients Building, 5 Beixiange Street, Xicheng, Beijing, 100053 China; 2 College of Traditional Chinese Medicine, Shandong University of Traditional Chinese Medicine, Jinan, China; 3 Department of Oncology, Guang’anmen Hospital of China Academy of Chinese Medical Sciences, 7 Floors of Inpatients Building, 5 Beixiange Street, Xicheng, Beijing, 100053 China

**Keywords:** Diabetes, Radiation therapy, Radiation pneumonia, Influence factors

## Abstract

Radiation pneumonia (RP) is a common adverse reaction to radiation therapy in patients with chest tumors. Recent studies have shown that diabetes mellitus (DM), which can cause systemic multisystem damage, specifically targets lungs, and the incidence of RP in patients with a history of diabetes is higher than that in other patients with tumors who have undergone radiotherapy. DM is an important risk factor for RP in tumor patients undergoing RT, and patients with DM should be treated with caution. This article reviews research on the clinical aspects, as well as the mechanism, of the effects of diabetes on RP and suggests future research needed to reduce RP.

Diabetes mellitus (DM) is a relatively common chronic metabolic disease [1]. The annual incidence of complications due to diabetes is increasing [2, 3]. Recent research has shown that diabetes is an important risk factor for radiation toxicity following radiotherapy for several tumors. Radiation-induced gastrointestinal and urogenital toxicity is more common in patients with DM [4, 5]. Radiation damage due to breast cancer treatment is also more common in patients with DM than in those without it [6].

Radiation therapy is a routine treatment for cancer after surgery. Radiation injury is one of the main adverse effects in patients who have undergone radiotherapy, and it is also the main limiting factor of the treatment [7]. When it occurs, radiation pneumonia (RP) usually presents within 1 to 6 months after completion of radiotherapy [8], with an incidence between 15 and 45% [4]. Patients with diabetes have shown a higher risk of radiation-induced damage [9], and many studies have indicated that lung tissue is a target for diabetes damage [10, 11], with an association between DM and RP. Glucocorticoids are the main drugs used to treat RP, but the administration of hormones can adversely affect blood glucose control and the insulin dosage required for diabetic patients. It also directly affects treatment of RP [12]. Therefore, some medical institutions exclude patients with severe diabetes from treatment with conventional radiation therapy [13]. The occurrence of RP in patients with breast tumors and DM has become a clinical concern. This article will review how DM affects RP based on clinical reports and mechanistic studies.

## Clinical evidence that DM affects RP

Hsia et al. [14] retrospectively analyzed clinical data from 118 patients with lung cancer and reported that DM itself was not associated with a higher risk of RP. Similarly, Orton et al. evaluated the correlation between DM and RP in 66 lung cancer patients and reported that, although patients who had lung cancer and DM showed a higher incidence of RP than patients with lung cancer alone, this incidence was not significantly different from that in patients without DM [9]. However, both of these studies were reported as abstracts only; full reports were unavailable. In China, there is a number of literature indicating that DM is an important risk factor for RP [15–17]. However, most of these studies were published in Chinese, and were performed with a small sample size and limited research scope. Therefore, their generalizability is uncertain. Kalman's team performed 424 computed tomography follow-up scans at 3, 6, and 12 months after treatment of 116 patients, including 27 diabetic patients, that received stereotactic radiation therapy (SBRT), and found that, in diabetic patients, tissue has denser and hazier than that of the non-diabetic patients, and that this was most obvious at 3 months after treatment. This indicated that lung injury after pulmonary SBRT is related to DM and most obvious at early stages after treatment [18]. This may be the first report to show an increase in the severity of RP after SBRT in diabetic patients. Due to the small sample size of the study, the period of lung injury should be interpreted cautiously. However, similar findings on the necessary caution of SBRT for diabetic patients had be reported. Kong et al. evaluated by univariate and multivariate analyses the effects of DM and DM-related serum factors (HbA1c and FBG) on the development of RP in 123 lung cancer patients that underwent chest radiotherapy, and confirmed that DM, HbA1c, and fasting blood glucose were significantly associated with the development of RP above grade 3. This was the first published report on the impact of DM-related serum factors on the development of RP [19]. This clinical evidence suggests that people with DM are more likely to develop RP than other people with lung cancer, and similar to the report by Kalman et al., that radiotherapy and post-treatment monitoring of lung cancer patients with DM should be conducted with caution. Collecting the retrospective data of 268 patients with NSCLC after radiotherapy and chemotherapy, Sefika et al. evaluated the relationship between RP risk and potential predictors through univariate and multivariate analysis, concluding that DM is an independent risk factor for RP [20]. The study identified diabetes as an independent risk factor for RP. However, there is a lack of research on the effect of diabetes classification on RP. At the same time, more large-scale randomized controlled trials (RCTs) are gain further relevant clinical evidence of the interactions between DM and SBRT.

## Mechanism underlying the effects of DM on RP

### Promotion of an inflammatory response

A 1993 article first described the relationship between tumor necrosis factor (TNF), inflammation, and insulin resistance in the journal *Science* [21]. At present, DM is considered a chronic inflammatory disease. Levels of inflammatory factors such as interleukin 6 (IL-6) and TNF-α are significantly increased in patients with DM, and type 2 DM (T2DM) is classified as a metabolic inflammatory disease [22, 23].

**Fig. 1:**
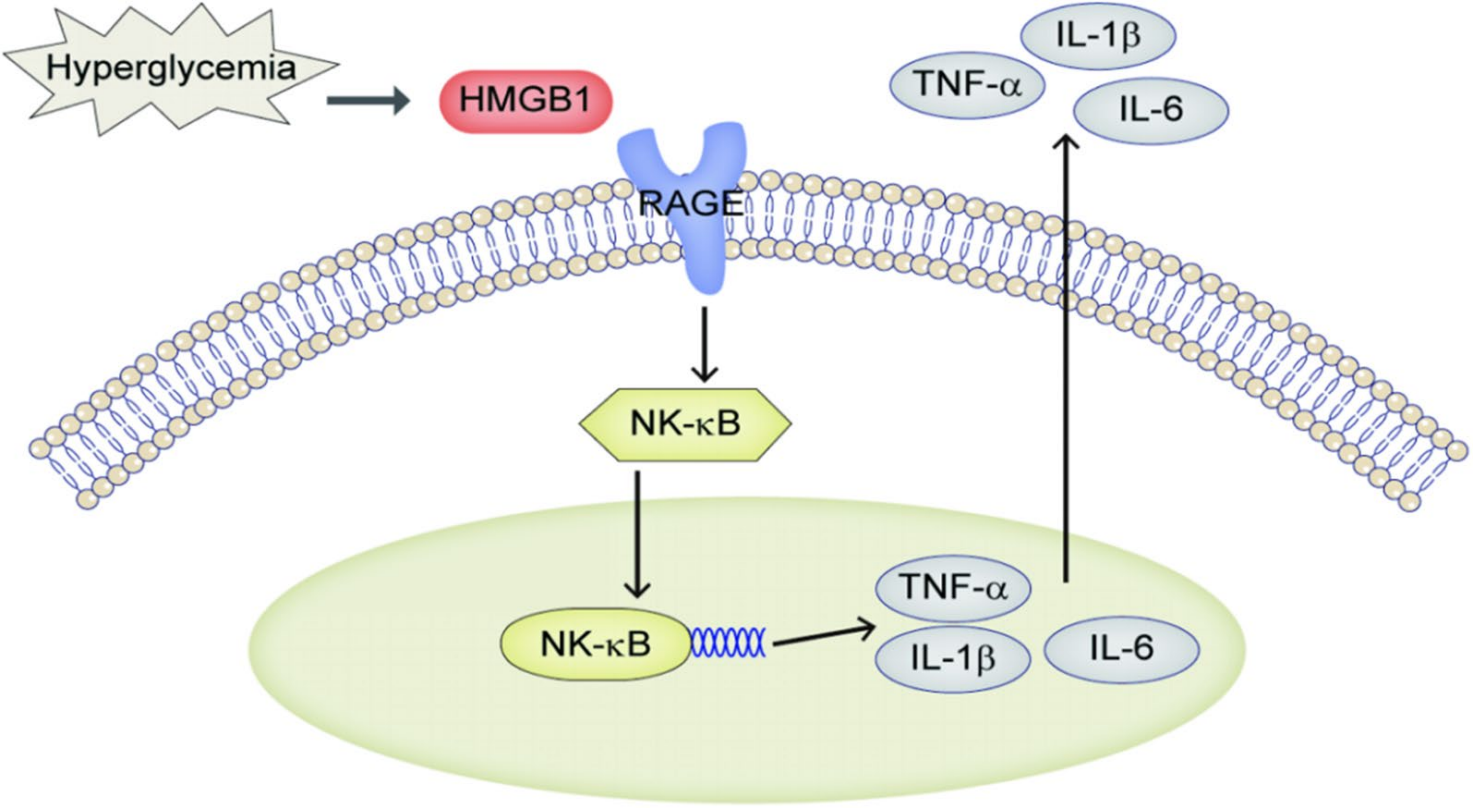
Diabetes induce NF-κB to promote inflammatory response. *HMGB*1 high mobility group 1 protein, *RAGE* receptor for advanced glycation endproducts, *NF-κB* nuclear facor kappa-B, *TNF-α* tumor necrosis factor α, *IL-6* interleukin 6; *IL-1β* interleukin 1β

**Fig. 2:**
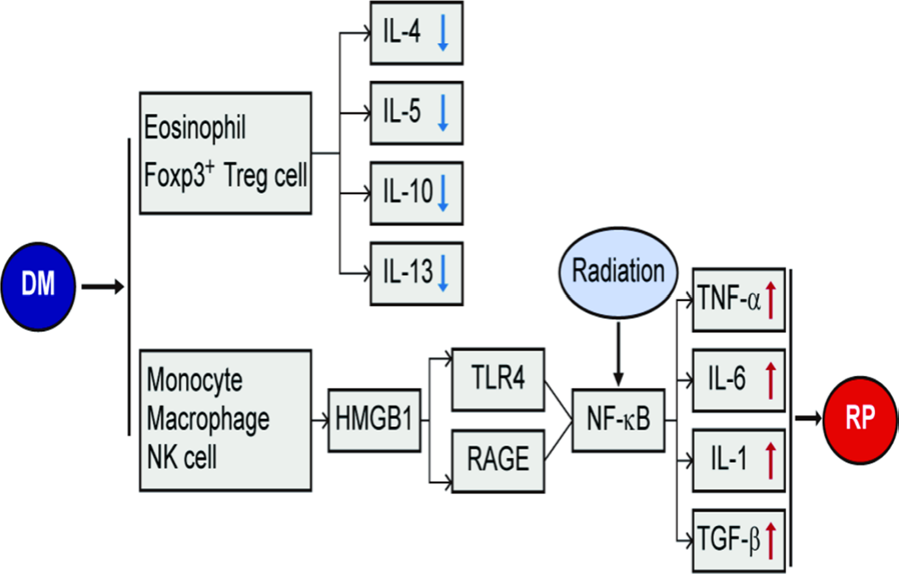
Inflammation and radiation pneumonia caused by diabetes. *DM* diabetes mellitus, *TLR4* toll-like receptors 4, *RP* radiation pneumonia

In the hyperglycemic environment of diabetic patients, anti-inflammatory cytokines such as IL-4, IL-5, and IL-13, which are typically maintained homeostatically in a microenvironment, lead to changes in the number and type of present cells [24, 25], presenting and releasing pro-inflammatory cytokines leading to chronic inflammation [26]. DM can also cause nuclear factor kappa-B (NF-κB) activation through receptor for advanced glycation endproducts (RAGE) [27], which is a member of the immunoglobulin superfamily of cell surface molecules, promoting TNF-α, IL-6, and IL-1β gene transcription and inflammation (Fig. 1) in the fat, muscle, and other tissues in our bodies [28]. Many reports have confirmed the important roles inflammatory cytokines such as IL-6, IL-8, and TNF-α play in mediating peripheral airway inflammation [29]. The "cytokine cascade" is currently recognized as the basis for RP pathogenesis [30]. In the early stages of radiation damage, changes in local inflammation and cytokine production can stimulate collagen synthesis in fibroblasts, leading to the development of pneumonia [31]. A hyperglycemic state activates inflammatory cytokines, aggravating lung tissue inflammation [32]. DM and its role in increasing inflammatory factors and exacerbating RP is gradually being recognized. Co-treatment of DM and RP should be the focus of future research (Fig. 2).

### Immune dysregulation

**Fig. 3:**
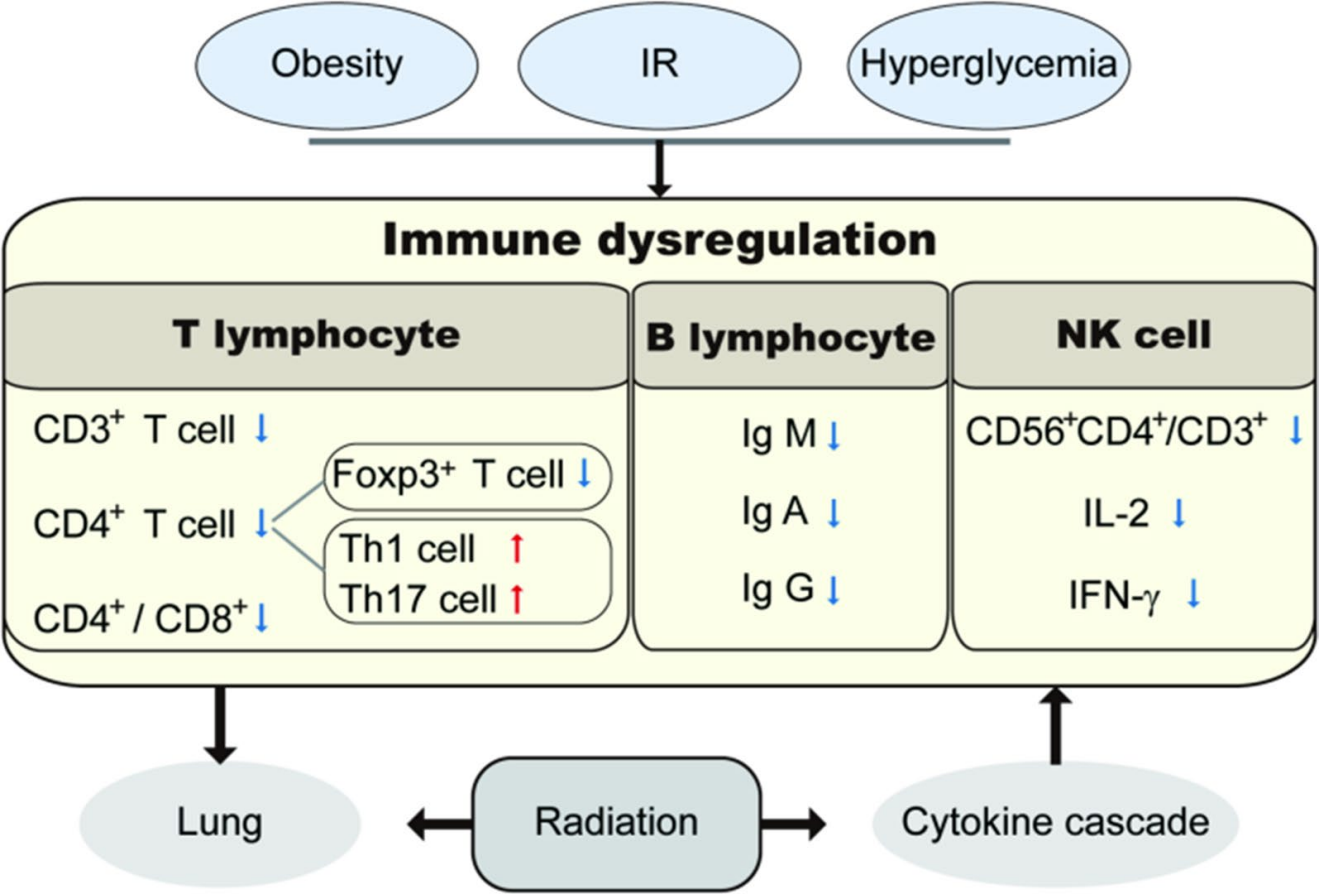
Immune dysregulation and radiation pneumonia caused by diabetes. *IR* insulin resistance, *Th1 cell* helper T cell 1, *Th17 cell* helper T cell 17, *IFN-γ* interferon- γ

In healthy individuals, immune responses are temporary. However, obesity, insulin resistance, persistent hyperglycemia, and other conditions can cause a chronic pathological immune imbalance that contributes to various diseases [33]. T2DM is an autoimmune disease, the accumulation of a large number of metabolites may cause immune dysfunction and damage immune function [34]. Changes in the proportions of T lymphocyte subsets can reflect changes in the body's immune status and are also closely related to the type and the degree of infection [35]. For example, the percentage of CD3 + and CD4 + T cells is lower in diabetic patients than in non-diabetics [36]. In addition, the hyperglycemic state of diabetic patients affects B lymphocyte function and humoral immunity, and results in IgM, IgA, and IgG antibody levels that indicate humoral immune deficiency [37]. T2DM can also impair NK cell function and increase the risk of infection and cancer [38, 39]. Immune imbalance is an important mechanism underlying infections concurrent with DM, because DM reduces a body’s resistance, and the higher incidence of vascular and peripheral neuropathy in diabetic patients [40]. Immune imbalance is also recognized in RP [41]. The immune system plays a role not only in the formation and development of RP but also prediction of this condition [42]. Diabetic patients have a disorder of glucose, fat, and protein metabolism, and the acidic environment in diabetic patients’ tissues reduces patients' cellular immune function, ultimately affecting the lungs [43] (Fig. 3).

### Oxidative stress

**Fig. 4:**
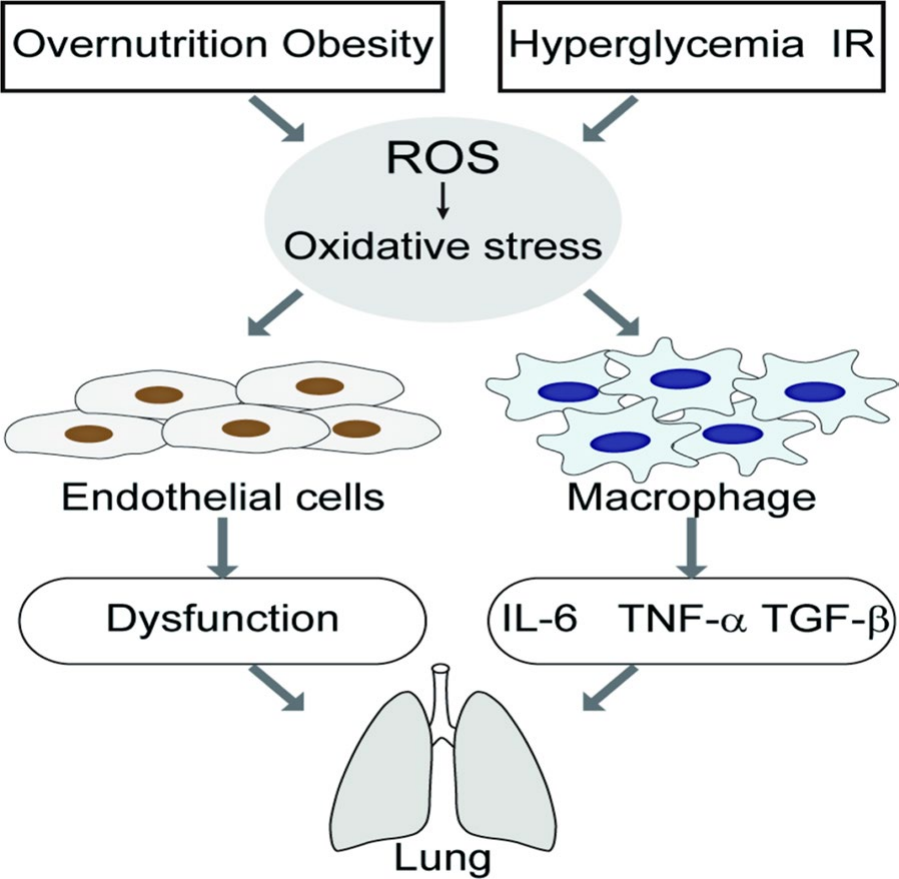
Oxidative stress and radiationpneumonia caused by diabetes. *ROS* reactive oxygen species

In DM, glucose and its metabolites react with hydrogen peroxide in the presence of iron and copper ions, forming hydroxyl groups during auto-oxidation and generating a large amount of reactive oxygen species (ROS). ROS, in turn, promote the development of complications, such as hyperlipidemia, end-stage renal disease and apoptosis of islet cells [44]. Insulin resistance caused by chronic hyperglycemia is also thought to be related to the induction of oxidative stress [45]. Akash [46] et al. studied the level of 8-hydroxydeoxyribonucleic acid-modified protein in Goto Kakizaki rats, a model of T2DM, and showed that hyperglycemia is the main underlying factor of pancreatic β-cell oxidative stress, the type of stress caused by glucose that underlies glucotoxicity. In 309 diabetic patients vs. non-diabetic controls, Charles Sturt et al. observed significantly increased levels of glycosylated hemoglobin, lipids, and oxidative stress biomarkers, and suggested that these findings support a link between long-term hyperglycemia in T2DM and hyperglycemia-induced oxidative stress [47]. Chronic persistent hyperglycemia has also been reported to contribute to microvascular and macrovascular complications that occur through a series of mechanisms involving oxidative stress and inflammation [47]. Oxidative stress injury is involved in initiating RP. The "cytokine cascade theory [48]" promotes the idea that ROS damages lung parenchymal cells, causing these cells to secrete a large amount of inflammatory cytokines, chemokines, profibrotic cytokines, and other factors that cause RP. In addition, Ma [49] et al. found that, with increased production of oxygen free radicals, the activity of oxidative stress-sensitive kinases and transcription cytokines in macrophages increased rapidly, leading to the production of large numbers of inflammatory cytokine such as TNF-α, IL-6, and transforming growth factor-β (TGF-β) (Fig. 4).

### Microvascular endothelial injury

As an important endocrine organ, the vascular endothelium produces cytokines can regulate vascular tension, vascular wall inflammation, and smooth muscle cell proliferation and adhesion, which can lead to vascular disease [50]. Recently, vascular endothelial injury has been recognized as a common cause of various DM vascular lesions [51], particularly at their initiation [52]. Animal experiments have shown that the basement membrane of alveolar epithelial and capillary endothelial cells in diabetic rats is significantly thickened, and some rats showed focal nodular changes that were very similar to the nodular changes in diabetic glomerular basement membranes [53]. Sustained hyperglycemia can also chronically damage vascular structures by promoting apoptosis of vascular endothelial cells [54], increasing the permeability of these cells [55] and regulating endothelial cell gene expression [56], eventually leading abnormal microvascular structure and function. Clinical studies have shown that in T2DM patients, the expression of some molecules that cause chronic structural damage to the vascular endothelium such as inflammatory factors (e.g., IL-6), vascular endothelial growth factor (VEGF), and angiopoietin (Ang) are upregulated, whereas the expression of molecules that exert vascular protective effects is significantly downregulated [57, 58]. The function and state of the endothelium depends not only on the extent of damage, but also on its ability to repair endogenously. In this regard, circulating endothelial progenitor cells (EPCs) play an important role because they can activate endothelial recovery [59]. In patients with diabetes, EPCs have been shown reduced ability to proliferate, adhere, and be incorporated into blood vessels [60]. In summary, DM causes endothelial dysfunction in many ways, but chronic damage to the structures and functions of vascular endothelial cells may be one of the main mechanisms underlying the development or aggravation of RP.

### Microvascular occlusion

Microcirculation is the most basic structural and functional unit in the blood circulation system. It regulates vasomotor smooth muscle contraction and affects blood flow through nerves and Bodily fluids. Microvascular occlusive lesions are a general term for microcirculation duct, blood flow, and functional obstructive lesions. They mostly occur in pathological conditions such as metabolic disorders and inflammatory reactions. They are characterized by slowed microvascular blood flow, increased vascular permeability, endothelial cell damage, and leukocyte adhesion and migration [61]. Lungs are extremely rich in microvasculature. Microvascular endothelial cell damage and increased vascular permeability caused by DM may lead to thickening of the fused basal lamina of alveolar septal epithelial cells and alveolar capillary endothelial cell basal lamina. In addition, some studies have found that diabetic microangiopathy is related to an imbalance in the fibrinolytic system [62]. Tissue-type plasminogenactivator (t-PA) is the initiating factor for the plasmin system [63]. It is regulated in the blood by plasminogen activator inhibitor type 1 (PAI-1). Patients with DM have long-term hyperglycemia, which causes an increase in PAI-1 that reduces the synthesis and secretion of t-PA, causing disorders of the fibrinolytic and coagulation systems, platelet activation, and microthrombosis, implicating peripheral nerve tissues and blood vessels corresponding tissue and organ lesions [64]. DM can cause microvascular occlusive lesions that lead to microcirculation disturbances in the lungs, which limit dissipation of local inflammatory reactions [65–67].

## Discussion

**Fig. 5:**
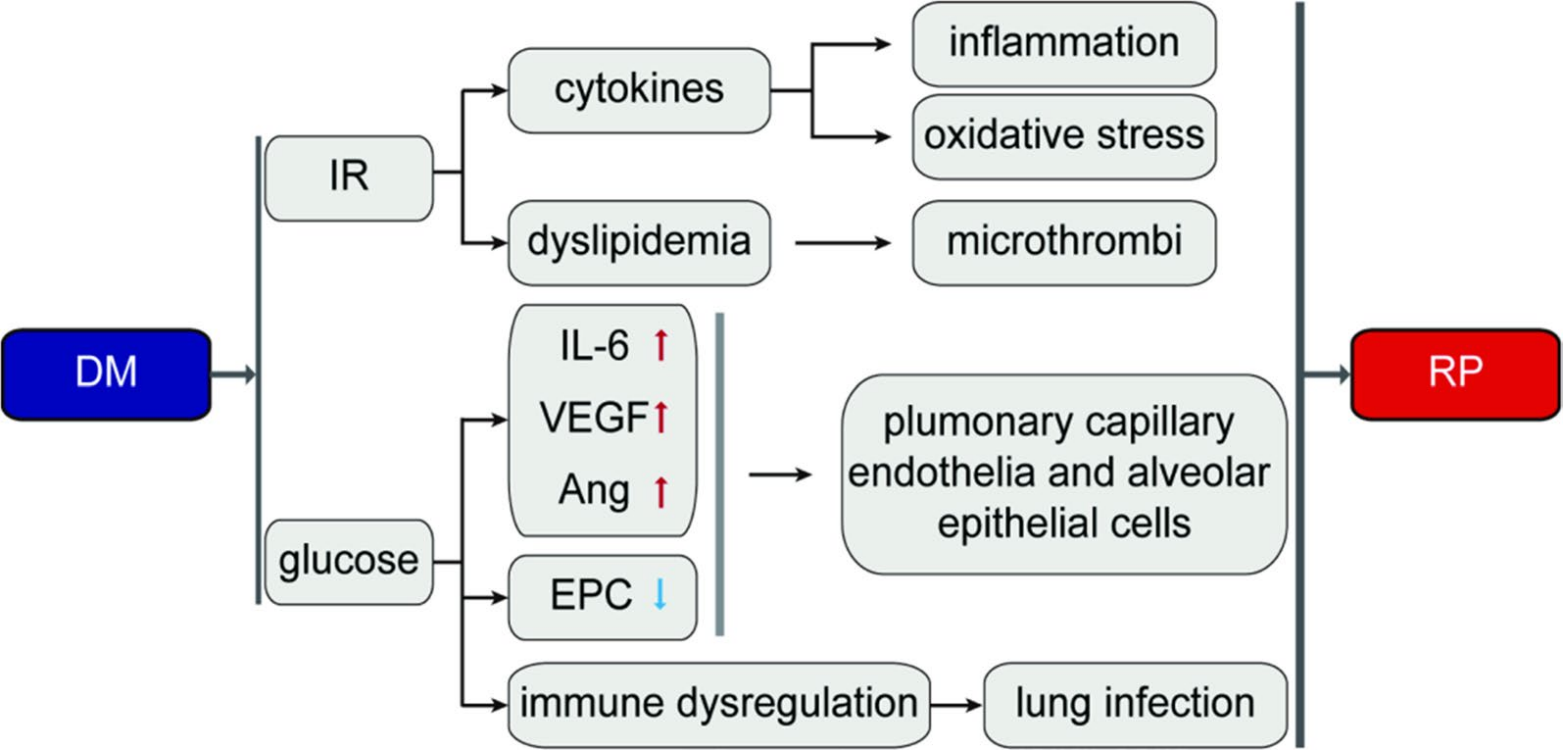
Effect of diabetes and other related factors on ration pneumonia. *VEGF* vascular endothelial growth factor, *Ang* angiopoietin, *EPC* endothelial progenitor cell

In the 1970s, Schuyler et al. [68] first proposed that lungs were a target organ of DM. In the past 40 years, evidence has accumulated that shows lung injury caused or exacerbated by high glucose can result in chronic progressive effects in lungs, poor quality of life, and limitations on the use of hormone therapies [69, 70]. The Third National Health and Nutrition Survey in the United Kingdom showed that lung function declines with impaired glucose tolerance [71], and that the relationship between T2DM and lung-related damage was significant [72].Through meta-analysis, Xiao-Jing Zhang et al. found that chronic lung disease or diabetes which lung cancer patients accompanied by were one of the important risk factors affecting the occurrence of RP [17]. Similarly, Sefika et al. found that DM was one of the important risk factors for the occurrence of RP by multivariate analysis and recursive zoning analysis [20]. In addition, the increase in X-ray volume and density of lung injury after radiotherapy was associated with the presence of diabetes mellitus and other clinical factors [18]. RP is one of the more common serious complications of chest radiotherapy [73]. At present, suitable and intensity-modulated radiation therapy is the main treatment for chest tumors, but the incidence of RP still reaches 30% of patients who undergo this treatment [74]. The pathophysiological processes of RP include inflammation, endothelial cell damage, microvascular circulation, and oxidative stress response [30]. DM is known to cause abnormal changes in the alveolar wall basement membrane, such as fibrinoid necrosis and fatty changes, thickening of the basement membrane of pulmonary capillary endothelial and alveolar epithelial cells, and increased permeability of blood vessel walls, exacerbating exudation at sites where inflammation occurs [75]. In addition, DM can result in microthrombi, leading to blood supply failure that cannot be repaired. Long-term hyperglycemia can also lead to cellular immune dysfunction and lymphocyte imbalances, resulting in low autoimmune function, susceptibility to various lung infections, and aggravated symptoms of RP [76]. In addition, patients with DM often experience autonomic neuropathy, parasympathetic activity with reduced bronchial adjusting tension resulting in reduced bronchial activity, bronchospasms, inflammatory exudation, retention of respiratory secretions, and reduced clearance by the airway ciliary mucosa, promoting conditions for local pathogen infection and reproduction and exacerbating RP [77] (Fig. 5).

DM is one of the most common endocrine diseases in the world. It is also the leading cause of death and disability worldwide [78]. Recent studies have shown that the prevalence of DM in lung cancer patients is higher than in the general population, and that patients with lung cancer should be more cautious about undergoing radiotherapy and adhering to post-treatment monitoring. DM is a risk factor for RP in cancer patients. Clinicians should fully consider this risk when developing radiotherapy plans and determining radiation doses. Maintaining fasting blood glucose levels within the normal range can reduce the occurrence of RP in patients with tumors and DM [79, 80]. However, there is still much that needs to be explored in interactions between DM and radiotherapy.

This article has several limitations. First, clinical studies reported in this article are mostly retrospective; therefore, there may be inherent biases. Specifically, we do not know how patients with DM faired long after treatment, with the potential for development of late-onset RP. Second, the sample sizes of these studies were relatively small. Third, patients and their tumors are heterogeneous. Despite these shortcomings, we believe that this article explores some important unresolved issues concerning the relationship between DM and RP.

## Conclusion

DM is an important risk factor for RP in tumor patients undergoing RT, and patients with DM should be treated with caution when undergoing this therapy.

## Data Availability

The datasets generated and/or analyzed in the current study are not publicly available due to state restrictions. Thus, information may compromise research participant privacy/consent but data are available from the corresponding author on reasonable request.

## Abbreviations

DM: Diabetes mellitus
RP: Radiation pneumonia
RCTs: Randomized controlled trials
TNF: Tumor necrosis factor
NF-κB: Nuclear factor kappa-B
RAGE: Receptor for advanced glycation endproducts
EPCs: Endothelial progenitor cells
PAI-1: Plasminogen activator inhibitor type 1
